# Circulating Tumor Cells from Surgical Manipulation Predict Recurrence and Poor Prognosis in Non-Small Cell Lung Cancer

**DOI:** 10.3390/jcm14062070

**Published:** 2025-03-18

**Authors:** Akikazu Kawase, Keigo Sekihara, Noriyuki Matsutani, Masafumi Yamaguchi, Yujin Kudo, Makoto Endo, Tetsukan Woo, Yuichi Saito, Noriyoshi Sawabata

**Affiliations:** 1 First Department of Surgery, Hamamatsu University of Medicine, Hamamatsu 431-3192, Japan; akawase@hama-med.ac.jp; 2 Department of Thoracic Surgery, Shin-Yurigaoka General Hospital, Kawasaki 215-0026, Japan; matsutan@med.teikyo-u.ac.jp; 3 Department of Thoracic Oncology, NHO Kyushu Cancer Center, Fukuoka 811-1395, Japan; yamaguchi.masafumi.wb@mail.hosp.go.jp; 4 Department of Surgery, Tokyo Medical University, Tokyo 160-8402, Japan; yjnkudo@gmail.com; 5 Department of Thoracic Surgery, Yamagata Prefectural Central Hospital, Yamagata 990-2292, Japan; m-endoh@ypch.gr.jp; 6 Department of Surgery, Yokohama City University, Yokohama 232-0024, Japan; tetsu.n.u@cotton.ocn.ne.jp; 7 Department of Surgery, Teikyo University School of Medicine, Tokyo 173-8605, Japan; k3699004@gmail.com; 8 Department of Diagnostic Pathology, Nara Medical University, Kahihara 634-8521, Japan; nsawabata@hotmail.com; 9 Department of Thoracic Surgery, Kawanishi City Medical Center, Kasai 666-0017, Japan

**Keywords:** non-small cell lung cancer, circulating tumor cells, prognosis, recurrence, surgical manipulation

## Abstract

**Background/Objectives:** In our previous multicenter prospective controlled study (UMIN000018602), we investigated the impact of surgical manipulation on circulating tumor cells (CTCs) in patients with non-small cell lung cancer (NSCLC). CTCs were detected after surgery in four patients (4/29, 13.8%), although CTCs were not present before surgery. These four patients had tumor cells leaked into their bloodstream by surgeons’ manipulation. We aimed to clarify long-term outcomes according to the presence of CTCs. **Methods:** Patients with cT1b-2N0M0 NSCLC scheduled for lobectomy were enrolled, based on the selection criteria of a consolidation-to-ground-glass opacity ratio (over 50%). Peripheral blood samples (≥3 mL) were collected before surgery (for pre-CTCs), during surgery, and immediately after pulmonary vein dissection (for post-CTCs). CTCs were isolated from these samples using ScreenCell^®^’s size-selective method. **Results:** From July 2015 to January 2016, 29 patients were enrolled, yielding paired pre- and post-CTC samples for all patients. Thirteen patients were pre-CTC positive, and post-CTCs were detected in 17 patients. Survival analysis revealed a statistically significant difference in recurrence-free survival between patients with and without post-CTCs (*p* = 0.043), while pre-CTCs status had no significant impact on recurrence (*p* = 0.226). Patients with post-CTCs had a significantly higher recurrence rate than those without (*p* = 0.043). Half of patients with post-CTCs but without pre-CTCs had recurrence within 5 years after surgery. **Conclusions:** Post-CTCs emerged as a significant predictor of recurrence following lobectomy; however, it could be possible for thoracic surgeons to prevent recurrence by improving surgical techniques for NSCLC patients with post-CTCs but without pre-CTCs.

## 1. Introduction

Non-small cell lung cancer (NSCLC) continues to carry a poor prognosis despite advances in treatment options [1]. Even with complete resection, the recurrence rate remains substantial [2,3,4]. The mechanism behind postoperative recurrence is thought to be as follows: microscopic cancer cells may already be circulating in the bloodstream at the time of complete resection, undetectable through imaging. Recurrence occurs when these cells lodge in major organs, such as the brain or liver, forming new tumor masses. These microscopic tumor cells, known as circulating tumor cells (CTCs), have been shown to correlate with poor prognosis across various cancers [5,6].

CTCs in the bloodstream are attracting attention as a biomarker for predicting cancer treatment effects and prognosis in various cancers. In NSCLC, past reports demonstrated that CTCs are useful for diagnosing lung cancer and predicting treatment effects [7,8,9,10,11]. These past studies investigated the presence or absence of CTCs in the bloodstream morphologically. The problem was that the detection rate of CTCs was low. This method mainly counts single cells using EpCAM, an antigen specific to epithelial cells, as an indicator, and its low sensitivity in the peripheral bloodstream was an issue that needed improvement. Past studies of CTCs mainly targeted pulmonary venous blood in lung cancer surgery cases [12,13]. As one way to solve this problem, we previously showed the impact of CTCs in peripheral bloodstream using ScreenCell^®^ [14].

We hypothesized that surgical manipulation could release CTCs from the NSCLC tumor into the bloodstream, potentially playing a significant role in recurrence. Based on this hypothesis, we previously conducted a multicenter study to examine the impact of surgical manipulation on CTC levels in NSCLC patients (UMIN000018602) [15]. In that study, pre-CTCs were present prior to surgical manipulation in 13 of 29 cases, with all these cases also showing post-CTCs. Among the remaining 16 cases without pre-CTCs, post-CTCs appeared after surgical manipulation in four cases. These findings suggested that surgical intervention may induce CTC release. After a 5-year follow-up, we report on the prognosis and recurrence status. This prospective study is the first to assess the prognostic impact of CTCs in this context.

## 2. Materials and Methods

### 2.1. Study Design

This multicenter prospective controlled study was approved by the Institutional Ethics Review Board (Teikyo University Review Board 15-010) and registered with the University Hospital Medical Information Network (UMIN000018602). All participants pro-vided written informed consent before study entry. Since no studies have been conducted on pre- and post-CTCs, a statistical prediction was not possible. Pre-CTCs were defined as CTCs which were detected in the blood sample from the radial artery before surgery. Post-CTCs were defined as CTCs which were collected in the blood sample after pulmonary vein dissection. Therefore, the study was considered evidence-generating with no hypotheses, and the number of patients was tentatively set. For a multicenter study, the number of patients enrolled annually was predicted to be 30. Institutions with at least 50 annual cases of curative surgery for primary lung cancer were invited to participate, and each institution was instructed to enroll a minimum of four patients to minimize technical variability of the ScreenCell^®^ between institutions. The primary endpoint of this study was the detection frequency of CTCs in the blood by the ScreenCell^®^ method. The secondary endpoints were the relationships between CTCs and pathological factors and CTCs and surgical manipulation. A concomitant retrospective study was the prognostic impact of CTCs.

### 2.2. Inclusion and Exclusion Criteria

In this study, patients who had been diagnosed with NSCLC by preoperative histological or cytological biopsy or by intraoperative rapid cytological or histological biopsy after lobectomy were enrolled. The stage of all patients was determined according to the 7th edition of the TNM classification of lung and pleural tumors. The study was designed for patients with cT1b-2 (2–7 cm) N0M0 NSCLC scheduled to undergo lobectomy. Eligible patients were between 20 and 80 years old, with an Eastern Cooperative Oncology Group (ECOG) Performance Status (PS) of 0 or 1, a proportion of consolidation to ground-glass opacity of over 50% on preoperative high-resolution computed tomography (CT), and patients who could tolerate lobectomy or higher based on preoperative respiratory function following two points: (i) predicted postoperative volume per second (FEV1.0) ≧ 800 mL and (ii) partial pressure of arterial oxygen (PaO_2_) ≧ 65 torr or arterial oxygen saturation (SaO_2_) > 90% (room air). Laboratory test results obtained within 14 days prior to enrollment had to meet all of the following criteria: (i) white blood cells (WBCs) ≥ 3000/mm^3^, ≤12,000/mm^3^; (ii) hemoglobin (Hb) ≥ 9.0 g/dL; (iii) platelet count ≥ 10 × 10^4^/mm^3^; (iv) aspartate aminotransferase (AST) ≤ 100 IU/L and alanine aminotransferase (ALT) ≤ 100 IU/L; (v) total bilirubin ≤ 1.5 mg/dL; and (vi) serum creatinine ≤ 1.5 mg/dL. Patients receiving corticosteroids or immunosuppressive drugs and those with severe disease (uncontrolled heart, lung, liver, or kidney disease or diabetes), synchronous or metachronous malignancies, or preoperative anticancer therapy were excluded from the study. Also, we excluded patients with active infections, including human immunodeficiency virus (HIV), hepatitis B virus (HBV), and hepatitis C virus (HCV) infections. Patients were excluded from this study if blood sampling was difficult for some reason, or even if the planned surgical procedure changed.

### 2.3. Blood Sampling

Blood samples for CTC testing were collected from a catheter inserted into the radial artery to monitor blood pressure during surgery, ensuring no additional invasive procedures were required for this study. Blood samples for pre-CTCs were collected before surgical operation in supine position. After confirming unresectable factors, including malignant pleural effusion or disseminated lesions, lobectomy with mediastinal lymph node dissection was performed by a thoracic surgeon under general anesthesia. Minimally invasive surgery (thoracoscopic surgery or thoracotomy with less than 8 cm skin incision) was performed according to procedures developed at each institution. The surgical approach was determined by surgeons based on the location and size of the tumor. In the operating room, a 22 or 20 G catheter was inserted into the radial artery under general anesthesia, and at least 3 mL of blood sample was collected in an EDTA-2Na-containing tube before surgery (for pre-CTCs measurement) and immediately after pulmonary vein (PV) dissection (for post-CTCs measurement). CTCs were extracted by the size-selective method using ScreenCell^®^ (ScreenCell CYTO R kit produced by SCREEN Holdings Co., Kyoto, Japan) using a 3 mL blood sample within 4 h of blood collection. The specimens were H&E-stained and prepared on glass slides with cover glass for microscopic analysis at each institution. The glass slides were stored in protective containers and transferred to the main research center. Three pathologists examined the glass slides blindly for the presence or absence of cancer cells (positive or negative). Cancer cells were confirmed by cytological examination, not by markers. The presence of even one cancer cell was considered as “positive”. Consistency of diagnosis by two of the three pathologists was used. All circulating cancer cells found were photographed with a digital camera and stored. The images of circulating cancer cells taken were sent to the principal investigator for confirmation. The presence or absence of circulating cancer cells were recorded using images taken with the digital camera and stored. Details on the diagnosis regarding the presence of CTCs are provided in our previous report [14].

### 2.4. Follow-Up Method

After surgery, a minimum follow-up period of 5 years was established at each institution. Chest X-ray was performed every 6 months. A chest CT scan was conducted every 6 months for the first two years post-surgery, followed by annual scans thereafter. Additional imaging, such as CT, magnetic resonance imaging (MRI), and/or positron emission tomography–computed tomography (PET-CT), was conducted depending on the patient’s symptoms. Pulmonary metastasis was a nodular shadow detected in the remaining lung. Differentiation from metachronous multiple lung cancer was based on the clinical course and the characteristics of the shadow, such as an increase in tumor markers. A definitive histological diagnosis was not made in all cases. Local recurrence was defined as recurrence in the ipsilateral lung, contralateral lung, bronchial stump, pleural dissemination, malignant pleural effusion, hilar lymph nodes, or mediastinal lymph nodes. All other recurrences were classified as distant recurrences.

### 2.5. Statistical Analysis

For the statistical analysis, the IBM^®^ SPSS Statistics version 26.0 (IBM, Armonk, NY, USA) software package was used. All *p*-values were two-sided, and *p*-values less than 0.05 were considered to represent statistically significant differences. Fisher’s exact test was used to analyze categorical variables, and Student’s *t*-test was used to analyze continuous variables. Survival curves were plotted using the Kaplan–Meier method, and log-rank tests were used to compare survival times. The zero time was the date of pulmonary resection. The endpoint of overall survival (OS) was defined as the date of death from any cause, and the last follow-up observation was censored when the patient was alive or lost to follow-up. The endpoint of recurrence-free survival (RFS) was defined as the date when recurrence was confirmed, or the date of death from any cause, and the last follow-up observation was censored when the patient was alive without recurrence or lost to follow-up.

## 3. Results

As previously reported, 31 patients from six centers were enrolled between July 2015 and January 2016. Two patients were excluded from the study due to the discovery of pleural dissemination during surgery, resulting in 29 patients included in the study. All patients were followed-up with for more than 5 years after surgery at each institution, with a median follow-up period of 5.1 years.

### 3.1. Patient Characteristics

The patients’ characteristics are summarized in Table 1. Pre-CTCs positivity was significantly more frequent in patients with pathological stage II or higher disease and those with pleural invasion. No other clinicopathological factors were significantly associated with pre-CTCs. Post-CTCs positivity was observed significantly more often in patients with pathological stage II or higher disease. No other clinicopathological factors were significantly associated with post-CTCs.

### 3.2. Detection of CTCs Before and After Pulmonary Vein Dissection

A cross table shows pre-/post-CTCs presence in Table 2. Pre-CTCs positivity and negative were detected in 13 and 0 patients, respectively. Post-CTCs positivity and negative were observed in 17 and 12 patients, respectively.

### 3.3. Postoperative Survival

**Figure 1:**
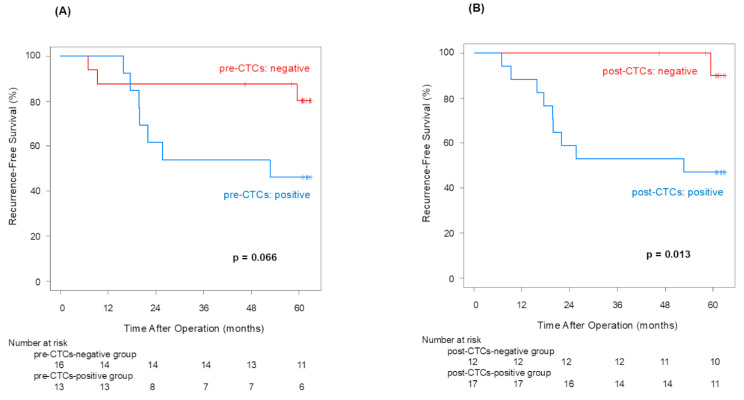
Recurrence-free survival curves for pre-CTCs (**A**) and post-CTCs (**B**).

The 5-year recurrence-free survival rates for the patients without or with pre-CTCs were 80.2% and 46.2%, respectively (*p* = 0.066; Figure 1A). The median recurrence-free survival term was 52.7 months in the pre-CTCs-positive group, and was not reached in the post-CTCs-negative group. The 5-year recurrence-free survival rates for patients with post-CTCs were significantly inferior to those in patients without (*p* = 0.013), at 90.0% and 47.1%, respectively (Figure 1B). The median recurrence-free survival term was 52.7 months in the post-CTCs-positive group, and not reached in post-CTCs-negative group.

### 3.4. Difference in Recurrence

After 5 years of follow-up, 9 of the 29 cases (31.0%) developed a postoperative recurrence. Patients without pre-CTCs developed a recurrence in 3 of 16 cases (18.8%), whereas patients with pre-CTCs developed a recurrence in 6 of 13 cases (46.2%). Although patients with pre-CTCs had a higher recurrence rate than those without, the difference was not statistically significant (*p* = 0.226). For post-CTCs, patients without post-CTCs developed a recurrence in 1 of 12 cases (8.3%), whereas patients with post-CTCs developed a recurrence in 8 of 17 cases (47.1%), with the latter showing a significantly higher recurrence rate (*p* = 0.043). There were 4 of 29 cases without pre-CTCs but with post-CTCs, of which 2 cases (50.0%) experienced a recurrence.

Regarding local recurrence, 2 of 16 cases (12.5%) without pre-CTCs and 2 of 13 cases (15.3%) with pre-CTCs developed local recurrence (Table 3). Patients with pre-CTCs had a similar local recurrence rate than those without (*p* = 1.0). For post-CTCs, local recurrence occurred in 1 of 12 cases (8.3%) without post-CTCs and in 3 of 17 cases (17.6%) with post-CTCs, but this difference was also not statistically significant (*p* = 0.622).

For distant recurrence, 2 of 16 cases (12.5%) without pre-CTCs and 7 of 13 cases (53.8%) with pre-CTCs developed a distant recurrence (Table 4). Patients with pre-CTCs had a significantly higher distant recurrence rate than those without (*p* = 0.041). In the case of post-CTCs, distant recurrence occurred in 1 of 12 cases (8.3%) without post-CTCs and in 8 of 17 cases (47.1%) with post-CTCs, showing a higher rate among those with post-CTCs, and this difference was also significant (*p* = 0.043). The details of recurrence sites are presented in Table 5, but no distinct relationship was observed between CTC status and recurrence site.

### 3.5. CTCs Induced by Surgical Manipulation

Four cases (25%) without pre-CTCs changed in CTCs status after surgery, converting the post-CTCs status into positive (positive convert group). In these cases, two of the four cases (50%) developed a recurrence. One case developed a recurrence in the mediastinum lymph node, the other developed bone metastasis. The characteristics of the patients in the positive convert group are shown in Table 6. All four cases showed no lymph node metastasis and no pleural invasion.

**Figure 2:**
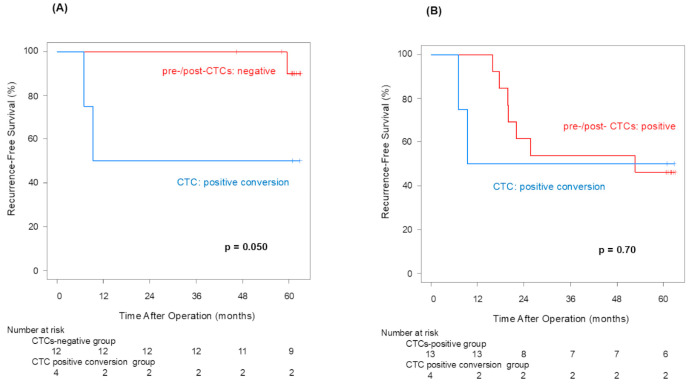
Recurrence-free survival curves for CTC positive convert group and pre-/post-CTCs-negative group (**A**), and for CTC positive convert group and pre-/post-CTCs-positive group (**B**).

The 5-year recurrence-free survival rates for the positive convert group and pre-/post-CTCs-negative patients were 50% and 90%, respectively (*p* = 0.050; Figure 2A). The 5-year recurrence-free survival rates for the positive convert patients and pre-/post-CTCs-positive patients were 50% and 46%, respectively (*p* = 0.70; Figure 2B). The median recurrence-free survival terms were 9.4 months in the positive convert group, 52.7 months in the pre-/post-CTCs-positive group, and not reached in pre-/post-CTCs-negative group, respectively. Except for one case that recurred in the distant period after 52 months, the two cases that recurred within 2 years were cases who showed positive conversion.

## 4. Discussion

In our previous report, the possibility of CTC dissemination into the bloodstream by surgical manipulation was demonstrated in cT1b-2N0M0 NSCLC patients (7). Among the 29 patients, 4 patients (13.8%) were considered to have post-CTCs positivity from a pre-CTCs-negative status, which could be induced intraoperatively by surgical technique. Hashimoto et al. investigated the relationship between surgery and CTCs by measuring CTC levels in blood drawn directly from the pulmonary vein before and after NSCLC surgery in 30 cases [15]. The median number of CTCs in the pulmonary vein increased significantly from 4 cells/2.5 mL before resection to 60 cells/2.5 mL after. Another study measured CTCs in peripheral blood taken before and after NSCLC surgery in 23 patients [16]. Among the 17 patients who did not have clustered pre-CTCs, 9 showed clustered post-CTCs in their peripheral blood. These findings, including our own, suggest that surgical manipulation for NSCLC may induce post-CTCs.

The presence of CTCs is a poor prognostic factor in various cancers [5,6] and has also been shown to predict a poor prognosis in lung cancer. Jin et al. reviewed studies on CTCs and prognosis in lung cancer, finding that CTC presence was significantly associated with worse prognoses in NSCLC (hazard ratio (HR) 2.11, 95% CI 1.63–2.73, *p* < 0.050) [17].

In our study, the pre-CTCs-positive rate was 44.9% and the post-CTCs-positive rate was 55.2%, a discrepancy which could suggest an intravascular influx of CTCs from the main tumor by surgical manipulation in 4 out of the 29 patients [12]. Although no statistically significant difference in OS in the pre-/post-CTCs groups was confirmed, the 5-year OS rate difference between patients with and without post-CTCs (100% vs. 76.5%) was equivalent to that observed with and without pre-CTCs (93.8% vs. 76.9%). And then, the CTCs-positive group showed poor OS in both pre- and post-CTCs (Appendix A). This finding suggests that CTC outflow may lead to poorer prognoses in NSCLC patients.

Pre-CTCs were not significantly associated with RFS (*p* = 0.066), but post-CTCs were significantly linked to poorer RFS (*p* = 0.013). Similarly to OS, the difference in the 5-year RFS rate between patients with and without post-CTCs (90.0% vs. 47.1%) was more significant than its difference between patients with and without pre-CTCs (80.2% vs. 46.2%), indicating that post-CTCs were associated with a poorer prognosis. Examining the site of recurrence, distant metastasis was significantly more frequent in cases that were positive for both pre-CTCs (*p* = 0.041) and post-CTCs (*p* = 0.043). In other words, this finding may suggest that post-CTC outflow strongly influences lung cancer recurrence. Here, we focus on patients who met the following conditions: pre-CTCs-negative and post-CTCs-positive (positive conversion group). In this group, it was highly possible that CTCs leaked from the tumor into the blood vessels due to surgeons’ manipulations. That could be why the RFS of the positive convert group was inferior to that of the pre-/post-CTCs-negative cases (*p* = 0.050). This result suggested that surgical manipulations have a possibility of worsening prognosis through CTCs.

Regarding the influx of cancer cells into the bloodstream due to surgical manipulations, Turnbull RB et al. reported in the 1960s that during colorectal cancer surgery, cancer cells may enter the bloodstream and cause recurrence [18]. Since this report, several studies on no-touch isolation techniques have been conducted, but no significant results have been obtained. Currently, prospective central vascular treatment is being investigated for colorectal cancer [19]. During surgery for NSCLC, primary tumors are subject to many physical influences, not only from the grasping and compressing the primary tumor during the operation, but also from the collapse and expansion of the lung due to separate lung ventilation, rapid needle biopsy, etc. Some have proposed that prior treatment of the pulmonary veins, which are the outflow tract, will prevent the release of cancer cells. But studies to date have not reached a clear conclusion [20]. Additionally, past reports suggested that surgical manipulations increase the levels of CTCs in pulmonary vein blood [13,21,22]. However, until now, the relationship between the increase in CTCs and prognosis was not clear [23,24,25]. We could prospectively examine prognosis for the first time. Examining the relationship between the increase in CTCs in the peripheral bloodstream and prognosis through intraoperative manipulation suggests the possibility of reducing metastasis and recurrence by devising improved surgical manipulations.

While surgically induced post-CTC outflow negatively impacted recurrence, OS was not significantly affected, likely due to advancements in NSCLC treatments, such as targeted therapies and immune checkpoint inhibitors, which may improve outcomes by controlling cancer progression after recurrence [26,27]. For recurrence rates, pre-CTCs did not show a significant difference (*p* = 0.266), whereas post-CTCs were significantly associated with increased recurrence rates (*p* = 0.043), supporting our hypothesis. Although we anticipated higher local and distant recurrence rates with post-CTC presence after surgical manipulation, this was not conclusively shown in this study.

This was a preliminary study with only 29 cases, and there were considerable limitations. In the near future, large-scale clinical trials enrolling more patients are needed in order to prove that surgical manipulation could have an important impact on long-term outcomes after lung cancer surgery. Also, our results suggested that it would be necessary to investigate the biological characteristics of CTCs in the future, such as analyzing surface ligand expression or performing DNA/RNA profiling for molecular markers. Surgical techniques the induced CTCs were observed in only four cases, and no trends in patient background, the characteristics of the CTCs, or the actual surgical procedures could be identified. This study suggests that surgical manipulation induces CTC release but did not specify how techniques varied across institutions. Future studies should provide details on surgical approaches (e.g., minimally invasive vs. open surgery) and their impact on CTC release. Nevertheless, the foundational data presented here could catalyze further research on CTCs and lung cancer prognosis, as well as the impact of surgical manipulation on lung cancer outcomes.

## 5. Conclusions

The presence of post-CTCs after PV dissection was associated with increased postoperative recurrence and significantly poorer RFS. However, no significant difference was observed in OS. Due to the small number of cases in this study, we were unable to identify specific effective surgical techniques. However, CTC monitoring may be a valuable tool in NSCLC management in the future.

## Figures and Tables

**Table 1:** Patients’ backgrounds.

		Pre-CTCs			Post-CTCs		
		Positive	Negative	*p*-Value	Positive	Negative	*p*-Value
Gender	female	5 (39)	10 (62)	0.27	7 (41)	8 (67)	0.26
	male	8 (62)	6 (38)		10 (59)	4 (33)	
Age	≤70	4 (31)	9 (56)	0.26	7 (41)	6 (50)	0.72
	>70	9 (69)	7 (44)		10 (59)	6 (50)	
Smoking index	<400	4 (31)	10 (62)	0.14	6 (35)	8 (67)	0.14
	≥400	9 (69)	6 (38)		11 (65)	4 (33)	
Tumor diameter	≤3 cm	7 (54)	7 (44)	0.71	8 (47)	6 (50)	1.0
	>3 cm	6 (46)	9 (56)		9 (53)	6 (50)	
Pathological stage	I	4 (31)	14 (87)	<0.01	7 (41)	11 (92)	<0.01
	II, III, IV	9 (69)	2 (13)		10 (59)	1 (8)	
Histology	adenocarcinoma	10 (77)	14 (87)	0.63	13 (77)	11 (92)	0.37
	non-adenocarcinoma	3 (23)	2 (13)		4 (23)	1 (8)	
Vascular invasion	negative	5 (39)	11 (69)	0.14	7 (41)	9 (75)	0.13
	positive	8 (61)	5 (31)		10 (59)	3 (25)	
Lymphatic invasion	negative	6 (46)	11 (69)	0.27	8 (47)	9 (75)	0.25
	positive	7 (54)	5 (31)		9 (53)	3 (25)	
Pleural invasion	negative	7 (54)	15 (94)	0.026	11 (65)	11 (92)	0.19
	positive	6 (46)	1 (6)		6 (35)	1 (8)	
Pulmonary metastasis	negative	12 (92)	16 (100)	0.45	16 (94)	12 (100)	1.0
	positive	1 (8)	0 (0.0)		1 (6)	0 (0)	
Total patients		13	16		17	12	

**Table 2:** Relationship between pre- and post-CTCs.

		Post-CTCs		
		Positive	Negative	Total
Pre-CTCs	Positive	13	0	13
	Negative	4	12	16
	Total	17	12	29

**Table 3:** The number of local recurrences in patients with pre-/post-CTCs.

		Post-CTCs		
		Positive	Negative	Total
Pre-CTCs	Positive	2/13	0/0	2/13
	Negative	1/4	1/12	2/16
	Total	3/17	1/12	4/29

**Table 4:** The number of distant recurrences in patients with pre-/post-CTCs.

		Post-CTCs		
		Positive	Negative	Total
Pre-CTCs	Positive	7/13	0/0	7/13
	Negative	1/4	1/12	2/16
	Total	8/17	1/12	9/29

**Table 5:** Relationship between CTCs status and recurrence site.

	Pre-CTCs		Post-CTCs	
**Recurrence Site**	Positive	Negative	Positive	Negative
Local recurrence				
Ipsilateral lung	2	1	2	1
Bronchial stump	1	0	1	0
Dissemination/malignant pleural effusion	0	0	0	0
Hilar lymph node	0	0	0	0
Mediastinal lymph node	2	2	3	1
Distant metastasis				
Contralateral lung	2	1	2	1
Supraclavicular lymph node	1	1	1	1
Brain	1	0	1	0
Liver	1	0	1	0
Bone	0	1	1	0
Adrenal glands	1	0	1	0
Total patients	13	16	17	12

**Table 6:** Patient background of CTC positive convert group.

Case	Gender	Age	Smoking	Tumor	Pathological	Histology	Vascular	Lymphatic
			Index	Size (cm)	Stage		Invasion	Invasion
1	M	75	880	4	IB	Sq	absent	absent
2	F	63	0	3.2	IIA	Ad	present	present
3	F	59	0	2.1	IA	Ad	present	present
4	M	60	820	3.2	IB	Ad	absent	absent

Ad: adenocarcinoma; CTC: circulating tumor cell; Sq: squamous cell carcinoma.

**Figure A1:**
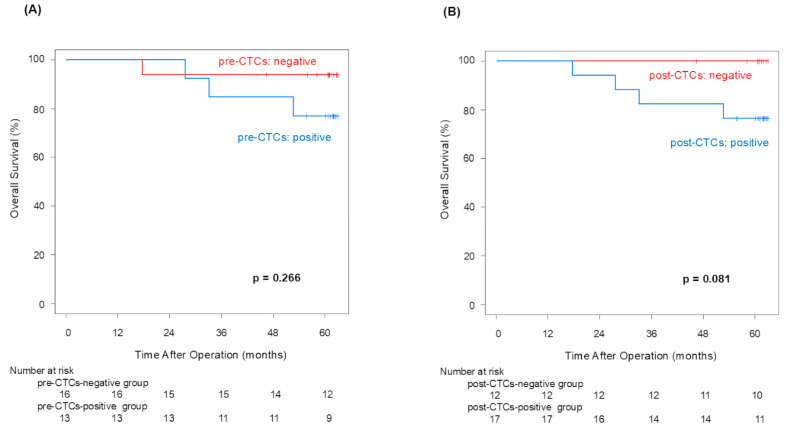
Overall survival curves for pre-CTCs (**A**) and post-CTCs (**B**).

**Figure A2:**
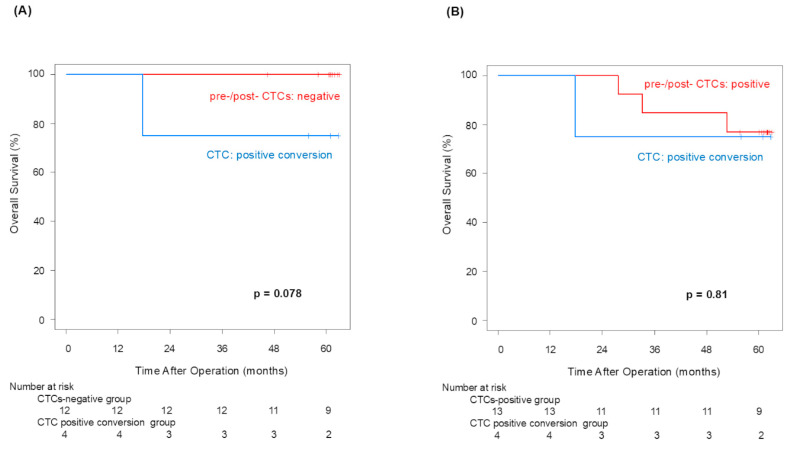
Overall survival curves for CTC positive convert group and pre-/post-CTCs-negative group (**A**), and for CTC positive convert group and post-CTCs-positive group (**B**).

## Data Availability

The raw data supporting the conclusions of this article will be made available by the authors on request.

## Abbreviations

ALT	Alanine aminotransferase
AST	Aspartate aminotransferase
CT	Computed tomography
CTCs	Circulating tumor cells
DNA	Deoxyribonucleic acid
ECOG	Eastern Cooperative Oncology Group
FEV1.0	Volume per second
Hb	Hemoglobin
HBV	Hepatitis B virus
HCV	Hepatitis C virus
HIV	Human immunodeficiency virus
HR	Hazard ratio
NSCLC	Non-small cell lung cancer
OS	Overall survival
PaO_2_	Partial pressure of arterial oxygen
PS	Performance Status
PV	Pulmonary vein
RFS	Recurrence-free survival
RNA	Ribonucleic acid
SaO_2_	Arterial oxygen saturation
WBC	White blood cell

## Appendix A

### Overall Survival

The 5-year overall survival rates for patients without or with pre-CTCs were 93.8% and 76.9%, respectively (*p* = 0.266; Figure A1A). The 5-year overall survival rates for patients with or without post-CTCs were 100% and 76.5%, respectively (*p* = 0.081; Figure A1B).

The 5-year overall survival rates for the positive convert group and pre-/post-CTCs-negative patients were 75% and 100%, respectively (*p* = 0.078; Figure A2A). The 5-year overall survival rates for the positive convert group and pre-/post-CTCs-positive patients were 75% and 77%, respectively (*p* = 0.81; Figure 2B).
